# Perlecan in Pericellular Mechanosensory Cell-Matrix Communication, Extracellular Matrix Stabilisation and Mechanoregulation of Load-Bearing Connective Tissues

**DOI:** 10.3390/ijms22052716

**Published:** 2021-03-08

**Authors:** Farshid Guilak, Anthony J. Hayes, James Melrose

**Affiliations:** 1 Department of Orthopaedic Surgery, Washington University, St. Louis, MO 63110, USA; guilak@wustl.edu; 2 Shriners Hospitals for Children—St. Louis, St. Louis, MO 63110, USA; 3 Bioimaging Research Hub, Cardiff School of Biosciences, Cardiff University, Cardiff, Wales CF10 3AX, UK; hayesaj@cardiff.ac.uk; 4 Graduate School of Biomedical Engineering, University of New South Wales, Sydney, NSW 2052, Australia; 5 Raymond Purves Laboratory, Institute of Bone and Joint Research, Kolling Institute, Northern Sydney Local Health District, Royal North Shore Hospital, St. Leonards, NSW 2065, Australia; 6 Sydney Medical School, Northern, University of Sydney at Royal North Shore Hospital, St. Leonards, NSW 2065, Australia

**Keywords:** perlecan: mechanosensation, elastin, fibrillin, PCM stabilization, tissue homeostasis, IVD biomechanics, mechanobiology, type VI collagen, meniscus

## Abstract

In this study, we review mechanoregulatory roles for perlecan in load-bearing connective tissues. Perlecan facilitates the co-acervation of tropoelastin and assembly of elastic microfibrils in translamellar cross-bridges which, together with fibrillin and elastin stabilise the extracellular matrix of the intervertebral disc annulus fibrosus. Pericellular perlecan interacts with collagen VI and XI to define and stabilize this matrix compartment which has a strategic position facilitating two-way cell-matrix communication between the cell and its wider extracellular matrix. Cues from the extracellular matrix are fed through this pericellular matrix back to the chondrocyte, allowing it to perceive and respond to subtle microenvironmental changes to regulate tissue homeostasis. Thus perlecan plays a key regulatory role in chondrocyte metabolism, and in chondrocyte differentiation. Perlecan acts as a transport proteoglycan carrying poorly soluble, lipid-modified proteins such as the Wnt or Hedgehog families facilitating the establishment of morphogen gradients that drive tissue morphogenesis. Cell surface perlecan on endothelial cells or osteocytes acts as a flow sensor in blood and the lacunar canalicular fluid providing feedback cues to smooth muscle cells regulating vascular tone and blood pressure, and the regulation of bone metabolism by osteocytes highlighting perlecan’s multifaceted roles in load-bearing connective tissues.

## 1. Introduction

### 1.1. Perlecan is a Modular Proteoglycan

**Figure 1:**
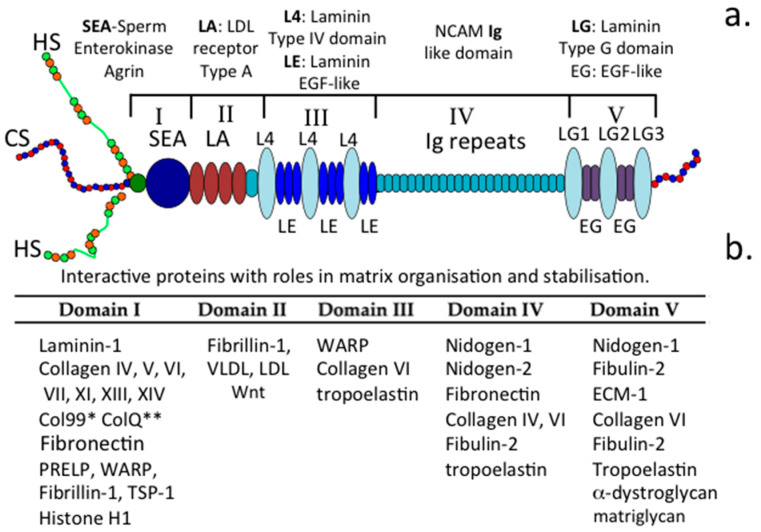
Schematic of the perlecan and its five modular domains (**a**). and their interactive ligands (**b**).

Perlecan is a large modular, multifunctional heparan sulphate (HS) proteoglycan (HS-PG) that is abundant in vascularized tissues but also occurs in poorly and non-vascularized connective tissues such as articular cartilage, intervertebral disc (IVD), meniscus, ligament and in tendon as a hybrid form, where at least one of its HS glycosaminoglycan (GAG) chains is replaced by a chondroitin sulphate (CS) chain [1,2] (Figure 1a). Smooth muscle cells (SMCs) synthesize a CS/HS hybrid form of perlecan whereas keratinocytes in epithelial tissues synthesize a form containing CS, HS and keratan sulphate (KS) [3], and endothelial cell perlecan is mono-substituted with HS. Mast cells synthesize perlecan species with smaller molecular weight core proteins [4] apparently arising from alternative splicing and/or protease cleavages in the immunoglobulin-rich domain IV, thus generating N and C terminal perlecan fragments of variable size. Some of these fragments act as functional PGs in their own right [5].

### 1.2. Perlecan’s Participation in Physiological Processes

Perlecan also has important regulatory roles in many physiological processes. Perlecan on endothelial cells in the lumen of blood vessels acts as a dynamic flow sensor [6] with detected shear forces regulating endothelial membrane polarization, cell proliferation, cytoskeletal organization and gene expression [7]. Furthermore, this is coupled with stimulatory biophysical forces that promote cell differentiation and tissue development [8]. Calcium signalling through transient receptor potential (TRP) channels in endothelial cells drives vasculogenic processes [9]. TRP channels also regulate the contractile properties of SMCs to regulate vasodilation and blood pressure [10]. Perlecan, is a mechanical biosensor in bone, detecting external loading through the identification of solute movement in the lacuno-canalicular space of the bone matrix [11,12,13].

### 1.3. The Role of Perlecan in Chondrocyte Mechanotransduction

Mechanotransduction—the conversion of a mechanical signal to an intracellular response—is a crucial process in the homeostatic maintenance of connective tissues such as cartilage. Deformation of cartilaginous tissues during normal daily activities exposes the chondrocytes to varying stresses, strains, hydrostatic pressure, interstitial fluid flow, electrokinetic effects and ionic changes that alter the local osmotic pressure [14]. Compression of the cartilage matrix causes water to be exuded from the tissue, while the negatively charged proteoglycans (PGs) are retained and attract positive counter-ions. This phenomenon results in fluctuations in the interstitial osmolarity of the ECM and pericellular matrix (PCM) [15,16] which can influence the physiologic activity of the chondrocyte [17,18]. In the last ten years, several ion channels, pumps or exchangers have been identified in the cell membrane of chondrocytes and shown to have important regulatory roles [19,20,21,22,23,24,25]. For example, the electrogenic Na+ /K+pump regulates Na+, K+ -ATPase and the resting potential of the chondrocyte [21]. Electrochemical gradients of Na+ and K+ are established and maintained by this active ATP-requiring pump [26,27]. The presence of Na+/K+, pump proteins in the chondrocyte cell membrane have been identified using immunological and autoradiographic techniques and multiple α, β, and γ, isoform subunits identified [28,29,30]. Proteomic studies on the chondrocyte ‘‘surface-ome’’, have confirmed the presence of multiple Na+/K+ pump isoforms [19]. As the ionic environment around chondrocytes clearly fluctuates during mechanical loading and relaxation cycles, chondrocytes have been shown to respond to changes in osmolarity with transient changes in intracellular Ca^2+^ that ultimately regulate the cells’ metabolic function and matrix synthesis [31]. Recent studies suggest that this mechano-osmotic transduction occurs via interactions of the ion channel transient receptor potential vanilloid 4 (TRPV4), a non-selective cation channel that is highly expressed in chondrocytes and interacts with the PCM [32]. The prominent localization of perlecan in the chondrocyte PCM stabilizes this pericellular environment and may regulate the maintenance of Ca^2+^ or Na+/K+ gradients in healthy chondrocytes, disruption of the PCM in arthritic chondrocytes may contribute to alterations in intracellular signaling and loss of chondrocyte homeostatis [32].

In addition to direct cellular signalling through mechanosensitive ion channels, growing evidence suggests that the ECM and PCM themselves can play important roles in transducing physiologic and pathologic mechanical signals in the cellular environment [33]. For example, fibroblast growth factor-2 (FGF-2), a major perlecan ligand, has been shown to serve as a mechanotransducer in the response of cartilage to trauma. Perlecan and FGF-2 are co-localised within the type VI collagen-rich PCM of articular cartilage. In damaged cartilage, chondrocytes release FGF-2 resulting in the activation of extracellular signal-regulated kinases (ERKs) and the induction of chondrocyte regulatory proteins including TIMP-1 and MMP-1 and 3 [34,35].

Biomechanical measurements conducted on single and groups of chondrocytes show that in cartilage, when a chondrocyte progresses from a single cell to a string of cells to a small and then larger cell cluster there is a progressive measurable decrease in stiffness in the PCM. This relaxation of the PCM makes the enclosed chondrocyte more amenable to influences from external stimuli from the ECM. The PCM also becomes more susceptible to proteolytic attack during the pathogenesis of OA [36]. In contrast to the wider, load-bearing ECM, the PCM contains relatively higher PG levels. Furthermore, small leucine repeat proteoglycan (SLRP) family members, such as biglycan and decorin, attach to the type VI collagen of the chondron [37] forming bridging structures between collagen and aggrecan-and HA networks. Perlecan, discussed in detail below, is also interactive with type VI collagen in the PCM [38] at all stages of joint development [39,40,41,42] contributing to the unique mechanical properties of the chondron [43]. This collagen-PG interconnectivity gives rise to coupled mechanosensory networks that extend out of the PCM into the inter-territorial matrix. These networks allow responsive remote sensing of the extracellular environment whereby environmental cues (e.g., perturbations in ECM) are fed back to the chondrocyte that responds appropriately to maintain cartilage homeostasis [44,45]. Collagen-PG networks contribute to the mechano-biology of articular cartilage in health and disease [46,47,48,49]. 

### 1.4. Perlecan Operates at Several Functional Levels in Load-Bearing Connective Tissues

**Figure 2:**
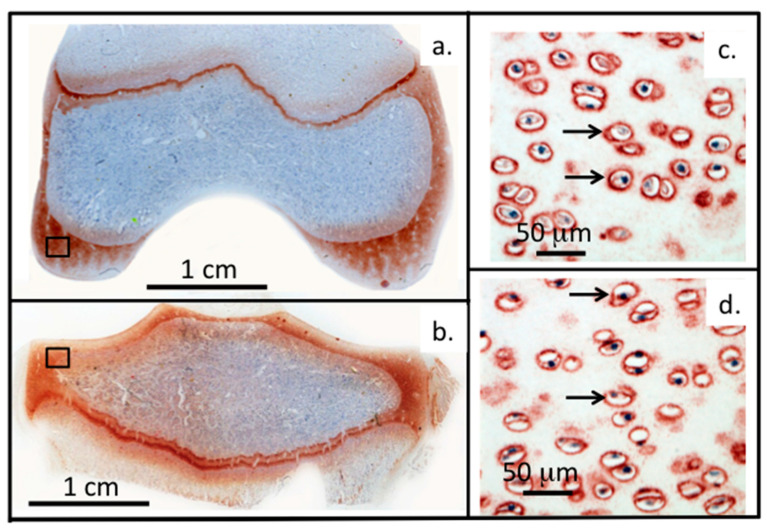
Perlecan and FGF-2 are prominent components of the PCM of chondrocytes. (**a**–**d**) Immunolocalisation of perlecan in an adult ovine stifle joint. Left panel: Macroscopic views showing femoral condyle (**a**) and tibial plateau (**b**). Right panel: High power views demonstrating perlecan’s pericellular distribution in femoral condyle (**c**) and tibial plateau (**d**). Figure (**a**–**d**) based on data originally published in [50].

Relatively few studies have examined how perlecan impacts on the biomechanical properties of connective tissues. Perlecan binds type VI collagen in the chondron surrounding chondrocytes in cartilage and has cytoprotective properties [38] (Figure 2). 

**Figure 3:**
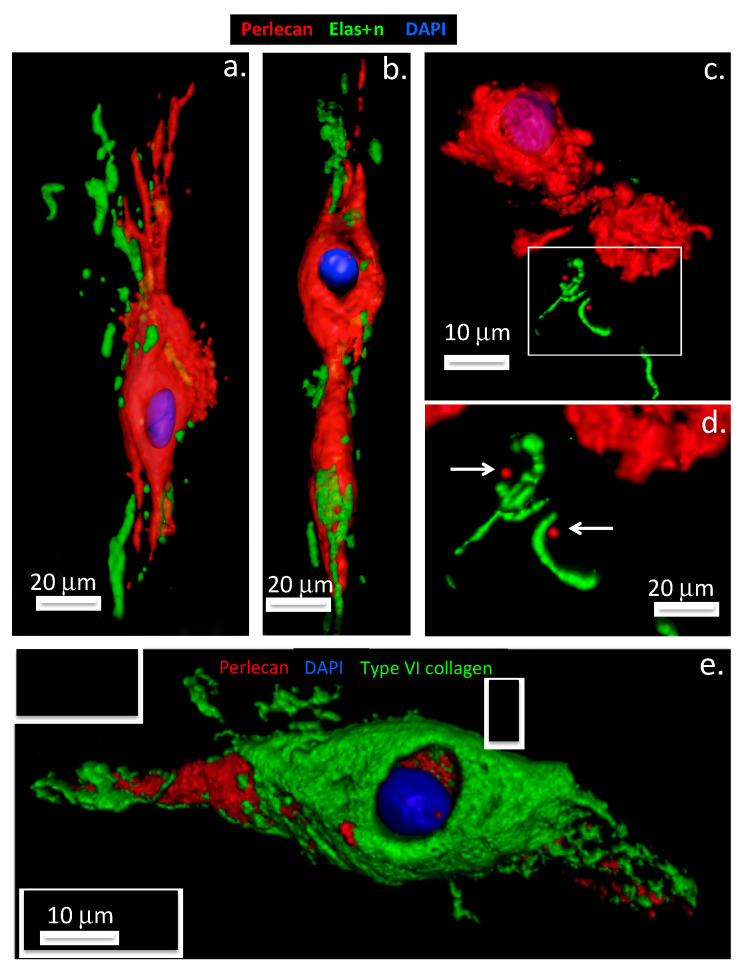
Perlecan promotes elastin formation by IVD cells. Three-dimensional surface rendered models of perlecan, elastin and type VI collagen immunolocalization in intervertebral disc tissues, based on confocal data originally published in [54,38]. Upper panel: Annulus fibrosus cells shown in (**a**,**b**), nucleus pulposus cells in (**c**,**d**). Secretion of co-acervated tropoelastin (green) is visible at the poles of the elongate, spindle shaped annulus fibrosus cells, perlecan label (red) extends outwards from the pericellular matrix compartment at the cell poles (**a**,**b**). In contrast, elastin is only sparsely distributed around NP cell clusters (**c**,**d**). Detail of the elastin labeling (boxed area in (**c**) is shown in (**d**) Arrows denote perlecan foci associated with elastin label in (**d**). Lower panel: The lower image (**e**) shows the overlapping distributions of collagen type VI (green) and pericellular perlecan label (red) within an annulus fibrosus chondron. Cell nuclei depicted in blue.

**Figure 4:**
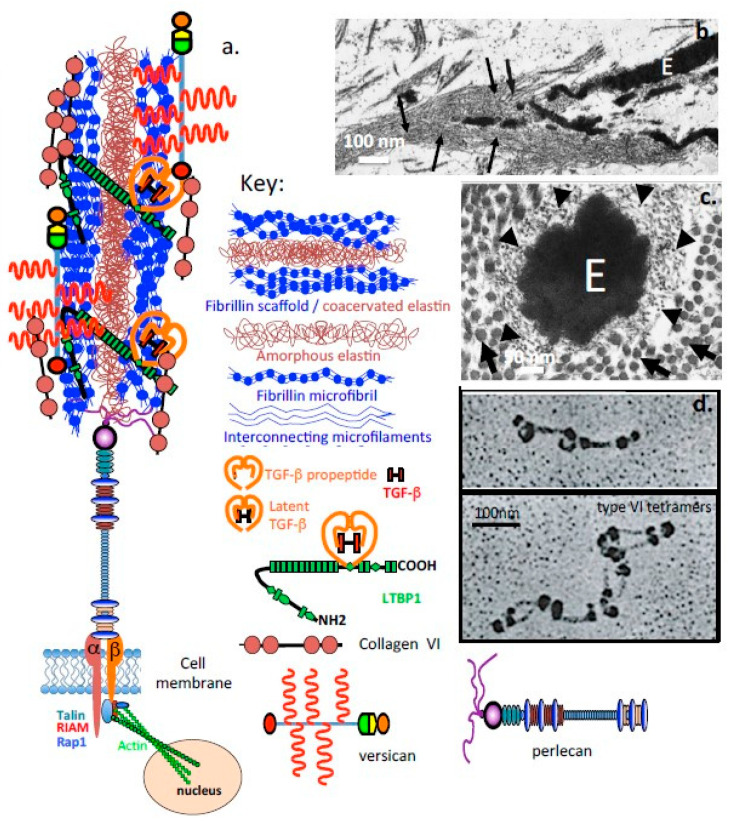
Schematic depiction of the elastic microbril, containing perlecan and fibrillin as functional omponents. (**a**) Schematic depiction of an elastic microfibril and its components. Electron micrographs of tannic acid-glutaraldehyde-fixed samples demonstrating the central amorphous elastin (E) within a microfibril in (**b**) longitudinal (magnification ×18,000) and (**c**) cross-section (magnification ×85,000); (**d**) Rotary shadowing images showing the characteristic interrupted globular domains in type VI collagen. Segment b, c reproduced from [55]. Segment d [56] under Open Access. Arrows in (**b**). depict microfibrillar material, E amorphous elastin. Arrowheads in (**c**). depict microfibrils in cross section, whilst arrows indicate collagen fibres in cross section.

Perlecan also promotes co-acervation of tropoelastin and is found associated with fibrillin-1, LTBP-2 (latent TGF-β binding protein 2) and a number of accessory elastic proteins promoting the formation of elastic fibrils at the cell surface [38,51,52,53] (Figure 3 and Figure 4). 

**Figure 5:**
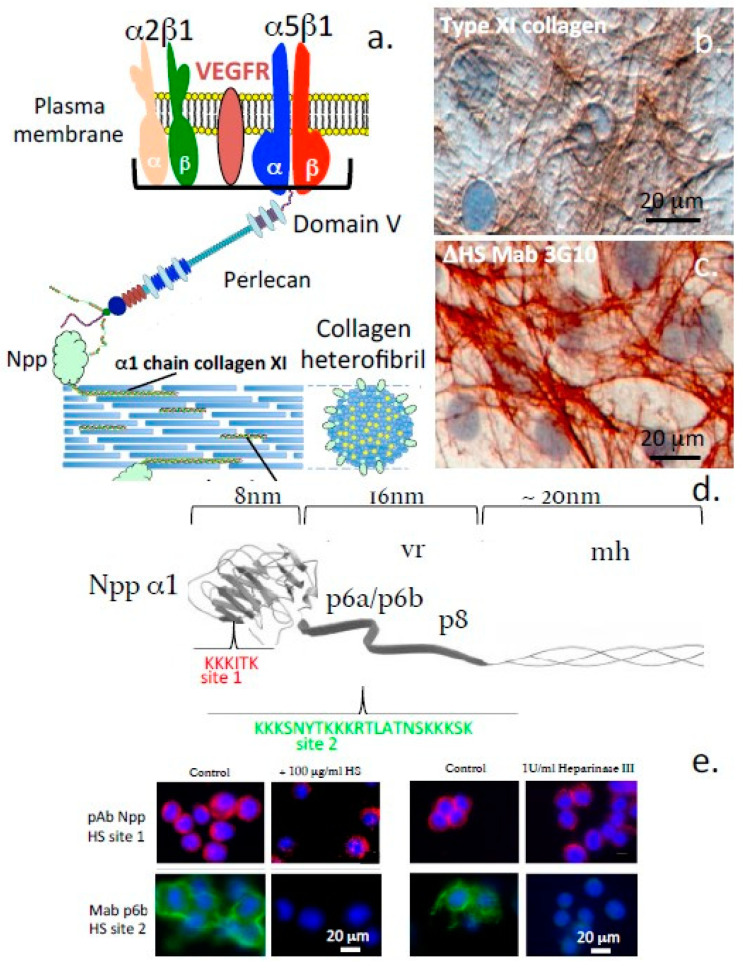
Perlecan interacts with cell surface integrins and type XI collagen in the PCM. (**a**) Diagrammatic schematic representation of the interaction of perlecan with cell surface integrins and the HSbinding sites of type XI collagen at the ovine disc cell surface. (**b**,**c**) Immunoperoxidase localisation of type XI collagen (**b**) and perlecan HS (**c**) in monolayer cultures of annulus fibrosus cells. (**d**) Schematic of the HS binding sites on the exposed Npp α1 domain of a type XI collagen heterofibril (**e**) Immunofluorescence localisation of type XI collagen in cultured chondrosarcoma cells showing pericellular HS-PGs. Segments a-c reproduced from [63]. Segments d and e reproduced from [64]. All plates in (**e**) are provided at the same magnification, 20 µm scale bar applicable to all segments.

Perlecan-elastin and perlecan-elastin-type VI collagen structures contribute to the viscoelastic material properties of the composite IVD tissues, particularly the annulus fibrosus (AF) [57,58,59,60]. Fibrillin-1 and elastin fibrils are also prominent components of translamellar cross-bridge structures in the IVD which are internal stabilizing structures that interconnect multiple, collagen-rich lamellar layers and provideresiliency to this tissue [61,62]. Perlecan co-localises with elastin in a number of tissues and with type XI collagen in the PCM of chondrocytes and IVD cells, stabilizing this structure and providing mechanosensory properties [63] (Figure 5). 

**Figure 6:**
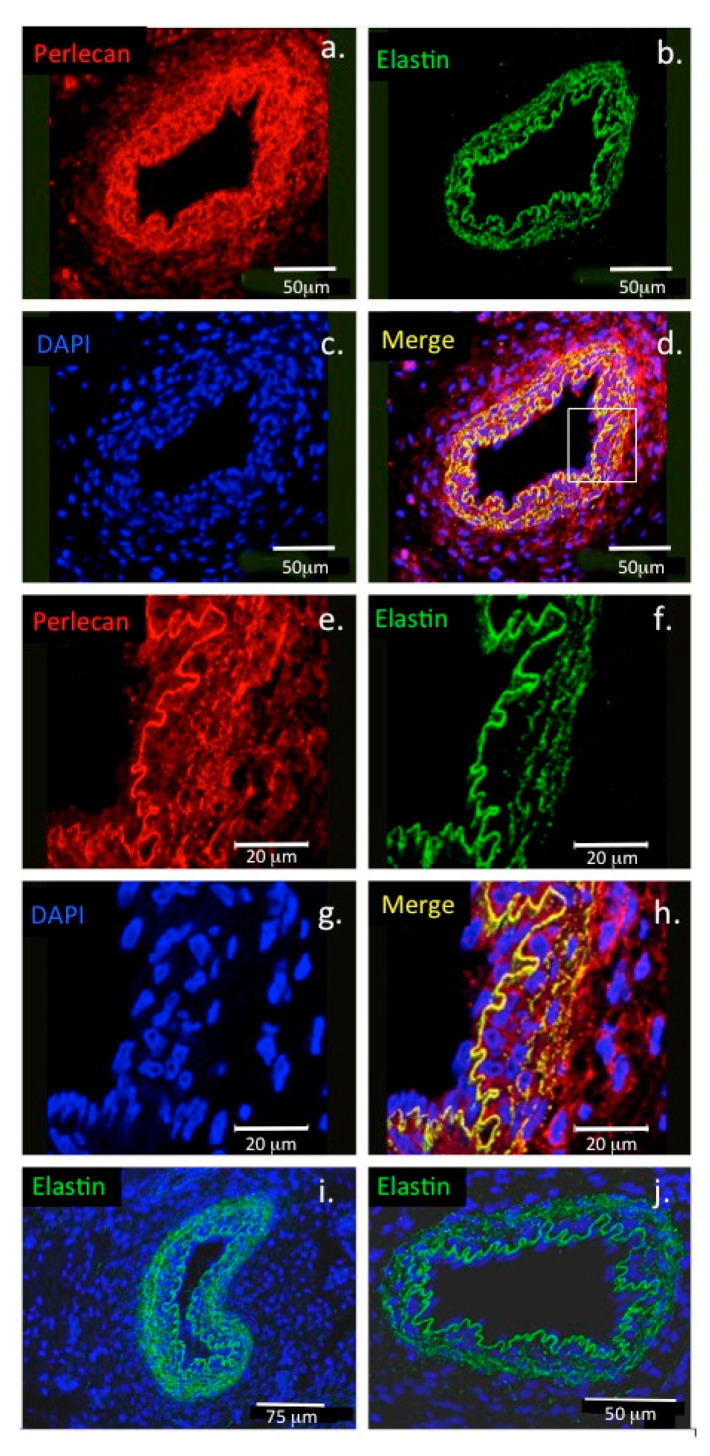
Colocalisation of perlecan and elastin in blood vessels. Confocal immunofluorescence localisations of perlecan and elastin in the basement membrane of a paraspinal blood vessel from a 14-week-old human foetal spinal specimen (**a**–**d**). Perlecan (red) and elastin (green) are colocalised (yellow) at the lumenal surface of the endothelial cell basement membrane of the blood vessel. The boxed area in (**d**) is shown at higher magnification in (**e**–**h**). (**i**,**j**) Elastin immunolocalisations are also shown in an additional paraspinal capillary (**i**) and in a venule (**j**). Cell nuclei depicted with DAPI staining (blue). Image reproduced from [54].

Perlecan interacts with α-dystroglycan in muscle tissues and has important roles in the assembly and function of the motor neuron neuromuscular junction (NMJ). It is a functional component of the specialized motor neuron synaptic endplate which interfaces with muscle fibres and controls muscular activity [65,66,67]. Perlecan also co-localises with elastin in endothelial cells in blood vessel basement membranes in vascularized tissues [54] (Figure 6).

From a biomechanical standpoint, the PCM appears to be defined similarly by the presence of type VI collagen as well as perlecan in both cartilage [43] and meniscus [68]. However, interior regions within the PCM containing both type VI collagen and perlecan exhibit lower elastic moduli than more peripheral regions that are rich in type VI collagen alone. The localization of perlecan to low modulus, interior regions of the PCM provides support for a potential role for HS and perlecan in the mechanical properties of the PCM as well as the process of mechanotransduction in cartilaginous tissues.

While the PCM is, in general, highly resistant to enzymatic digestion by chondroitinase, aggrecanase, or hyaluronidase [69], enzymatic removal of HS chains from perlecan with heparinase III results in increased elastic moduli in the PCM, specifically within interior regions of the PCM that are positive for both perlecan and type VI collagen. Interestingly, heparinase III digestion has no effect on the micromechanical properties of the ECM. These findings suggest that perlecan plays a biomechanical role in the PCM compartment, potentially serving as an additional “integrator” of the various matrix proteins focused on this region. Indeed, the fact that intact chondrons can be isolated from mice genetically lacking collagen VI suggests that additional matrix macromolecules such as perlecan are required to maintain the structural integrity of the PCM.

### 1.5. The Functional Attributes of Perlecan’s Five Domains

#### 1.5.1. Domain-1

Perlecan interacts with several collagens and cell adhesive and structural glycoproteins aiding in the organization and stabilization of the ECM and cell-ECM interface. Many of the collagens (e.g., collagen types IV, XIII, XIV) interact prominently with perlecan within the basal lamina of blood vessels, the blood–brain barrier, membranous structures surrounding nerves in the peripheral and central nervous systems (PNS/CNS), the meninges surrounding brain and spinal cord, and around nerve bundles. Some of the collagens have specific HS binding sites (e.g., collagen types V, XI) and can be incorporated into heterofibrils with collagen types I and II and this may contribute to microfibril and fibril bundle assembly in tendons. Fine type XI collagen fibres localize with perlecan in the PCM around cells and may have stabilizing roles to play in this region [63]. Other ECM proteins, including PRELP (proline/arginine-rich end leucine-rich repeat protein), WARP (von Willebrand factor A domain-related protein), laminin, thrombospondin and fibronectin also have HS binding sites which contribute to the networking of these components in the ECM. Col 99 is a transmembrane collagen that is interactive with perlecan found in *Caenorhabditis elegans.* CollQ, a collagen-tailed form of acetylcholinesterase is anchored in the NMJ by perlecan and is essential for motor nerve functional properties at the nerve-muscle interface which regulates muscular movement and is wired into the CNS allowing co-ordination of the activity of different muscle groups to control skeletal movement. PRELP is a member of the SLRP family and acts as an anchorage point for perlecan in many basement membranes [70]. WARP is another perlecan-interactive protein that has stabilizing properties in specialized membranous tissues surrounding nerves [71]. Perlecan interacts with collagen type VI in the PCM of tensional and weight bearing connective tissues (e.g., cartilage, IVD and meniscus) where it provides compliant and cyto-protective properties [38,43]. Perlecan also aids in the co-acervation of tropoelastin at the cell surface and in the assembly of a fibrillin scaffold around these nascent molecular structures to form elastic microfibrils which provide mechanosensory properties to the cells they are attached to [54].

#### 1.5.2. Domain II

Perlecan domain II binds fibrillin-1, lipoproteins, and lipids. Excessive build up of these components on endothelial cells at the luminal surface of blood vessels contributes to atherosclerotic plaque formation. This is balanced by the ability of perlecan to clear lipids from the blood stream and their endocytosis by the vessel endothelium. Domain II acts as a low-density lipoprotein (LDL) receptor and binds Wnt and hedgehog proteins which are lipid-modified proteins of limited solubility in aqueous media. This aids in the transport of these proteins and also facilitates formation of biochemical gradients that drive tissue development and morphogenesis.

#### 1.5.3. Domain III

WARP binds to perlecan domain III. WARP has a restricted expression in the permanent cartilages and a distinct subset of basement membranes in peripheral nerves, muscle, and CNS vasculature. Besides binding to perlecan, WARP also binds to type VI collagen, and may act as a bridging structure between protein networks in connective tissues [71]. Collagen type VI also binds to perlecan domain III, as does tropoelastin, suggesting that this domain may promote the formation of elastic microfibrils with mechanosensory functions.

#### 1.5.4. Domain IV

Human perlecan domain IV contains 21 immunoglobulin G repeats, sharing homology with NCAM, and it also contains a novel peptide sequence that supports cell adhesion, spreading and activation of the FAK (focal adhesion kinase) cell signaling pathway [72]. Domain IV is the largest of perlecan’s domains and it acts as a template for the attachment of a number of ECM-stabilising proteins including, nidogen-1 and 2, fibronectin and collagen types IV and VI, thus it has a prominent role to play in ECM stabilisation [73,74]. Domain IV is also susceptible to degradation by a number of MMPs and serine proteases. The domain IV fragmentation pattern evident in various stages of prostate cancer may have utility as a biomarker of disease progression [75]. It has also been suggested that degradation of domain IV converts it from a tumour-inhibitory form to one which is ‘tumour-friendly’ [76].

#### 1.5.5. Domain V

Domain V is composed of three laminin G domains which are each separated by two EGF-like domains. Domain V interacts with a number of ECM-stabilising proteins (nidogen-1, fibulin-2, ECM-1, collagen type VI), however the major interest of domain V lies in its angiogenic properties which have been proposed as a prospective therapeutic to treat ischaemic stroke [77,78]. Fragments of domain V containing the LG1-LG2 domains or LG3 domain can block VEGFA (vascular endothelial growth factor A) activity or binding to α2β1 integrin providing anti-angiogenic activity. A GAG attachment site has also been identified in the LG3 domain but it is not always occupied [79].

### 1.6. Fibrillin-1 Stabilizes Tissues and Provides Essential Functional Properties

Fibrillin-1 is a fibrillar 350 kDa calcium-binding protein that has a widespread distribution in connective tissues occurring in assemblies of 10–12 nm microfibrils. Fibrillin 1-3 contain two types of disulfide-rich motifs, the calcium-binding epidermal growth factor-like (cbEGF) and transforming growth factor beta binding protein (TGFBP)-like domains as dominant functional determinants. Elastin and fibrillin-1 are building blocks for elastic fibers that support the mechanical stabilisation of tissues [80]. Fibrillin microfibrils provide biomechanical properties to tissues endowing them with limited elasticity and durability and these act as templates for elastin deposition during elastic fibre assembly [81]. Recent evidence suggests that tropoelastin initially undergoes assembly within the cell or at unique assembly sites on the plasma membrane to form elastin aggregates (Figure 3a,b), which, when outside the cell, transfer to fibrillin scaffolds-tensional forces transmitted through these structures then help to shape the growing microfibril [82] (Figure 4c,d). The recent identification of intracellular perlecan in IVD cells suggests potential roles in this aggregation process, and is consistent with roles ascribed to HS in the initial stages of the co-acervation of tropoelastin [83,84]. Knock-out studies in mice have also shown that accessory proteins such as fibulin-4, -5 and LTBP-4 also play crucial roles in the elastogenic process [85]. Elastic microfibrils also represent a storage niche for growth factors in tissues, mutations in fibrillin cause Marfan syndrome and short-stature pathologies due to dysregulated growth factor signalling. Thus while elastin-fibrillin microfibrils have mechanosensory roles in tissues they also have major regulatory functional roles in growth factor regulation [86] (Figure 4b).

### 1.7. Interactive Properties of Perlecan, Assembly of Elastic Microfibrils and Their Functional Attributes

Confocal studies have demonstrated that perlecan in the PCM of AF cells promotes the co-acervation of tropoelastin synthesised by this cell type [52,53,54]. A quartz crystal microbalance study also demonstrated the de novo assembly of elastin on a quartz crystal coated with perlecan in vitro [54]. Fibrillin-1 fibrils are also assembled at the AF cell surface surrounding the coacervated elastin to form a composite elastin-fibrillin fibril as found in many other connective tissue cell types (Figure 4c,d). These elastic fibrils are found co-distributed with collagen fibrils including type VI collagen and versican and are also attached to cell-associated perlecan in the annular tissues. Cells of the AF and nucleus pulposus of the IVD possess a PCM rich in type VI collagen, similar to that of articular cartilage, which plays a crucial role in the transmission of mechanical signals from the surrounding environment of the cells [87,88,89] (Figure 3). The AF undergoes both tension and compression in the composite IVD tissues. Perlecan also colocalizes with elastin and fibrillin-1 in blood vessels [54]. These observations are consistent with published studies identifying HS as a component of early microfibril assembly [84] and the linkage of fibrillin fibril bundles with other connective tissue networks by versican [90]. Fibronectin serves a similar role in the assembly of elastic fibrils with other cell types [91,92] and governs the deposition of LTBP in tissues [93]. Elastic microfibrils are not only structural determinants in tissues but also play mechanosensory roles that allow cells to maintain tissue homeostasis [94,95]. Elastic microfibrils are also repositories for growth factors that have roles in tissue development and morphogenesis [96,97]. The fibrillin microfibril scaffold contains a number of associated proteins and growth factors, e.g., transforming growth factor-β (TGF-β), bone morphogenetic proteins (BMPs) etc, thus these are mechano-responsive structures capable of feeding mechanosensory signals back to cellular receptors through the fibrillin fibril extracellular network [97,98,99]. This complex interchange of information regulates tissue homeostasis and development and growth [94,95,96]. Abnormal functioning of this system is evident in Marfan’s Syndrome and other pathological conditions of short stature.

### 1.8. The Pericellular Matrix, a Dynamic Cell-Matrix Interface

The PCM is a key region in tissues, representing an interactive interface between cells and their biomechanical micro-environment [100]. The PCM also protects cells in hostile weight-bearing environments [63,101]. Perlecan plays essential cyto-protective roles in the PCM as perlecan knockdown severely impacts PCM organisation, composition and functional properties [102] (Figure 2c,d). A structurally impaired PCM results in mechanical overload to the chondrocyte and cell death [103]. Loss of spatial organisation and destruction of the PCM in articular cartilage occurs in osteoarthritis (OA) [104] and is a hallmark of the degenerative processes that lead to the development of this joint disease [105,106]. Micromechanical mapping of early changes in the chondrocyte PCM in OA, atomic force microscopy (AFM) [107,108] and multiphoton microscopy [109] have demonstrated the importance of the correct structural organisation and mechanotransductive pathways in chondrocyte regulation in the maintenance of tissue homeostasis [110,111,112]. Perlecan interacts with collagen types VI and XI and a number of cell adhesive and structural glycoproteins to stabilise the PCM of the chondrocyte [32,38,63,113], and enzymatic digestion of perlecan alters the biomechanical properties of its PCM [43]. Perlecan is also a component of basal laminar structures and blood vessels in the synovium and paraspinal tissues (Figure 6). Confocal reconstructions of AF cells and articular chondrocytes labelled for perlecan and collagen type VI show the overlapping pericellular localisations of perlecan and collagen type VI within the chondron structure (Figure 3e). While perlecan has a pericellular distribution around chondrocytes in the rudiment and growth plate cartilages it also has an extracellular distribution in these tissues during morphogenetic change with tissue development. This contrasts with the highly focussed PCM distribution of perlecan around articular chondrocytes of mature cartilage tissues, i.e., with no detectable perlecan within the interstitial and inter-territorial ECM, as once formed, these permanent cartilages, once formed, do not undergo further morphogenesis (Figure 2c,d). Perlecan is also found in translamellar cross bridge structures within the IVD having a polarized distribution at the ends of strings of fusiform AF (Figure 3) and ligament cells and being surrounded by linear arrays of fibrillar collagen. Furthermore, in the meniscus, perlecan has PCM and ECM distributions similar to that of chondrocytes found in growth plate cartilage [114]. Thus, as the precise spatio-temporal distribution of perlecan differs in particular cartilaginous tissues, its realms of influence varies accordingly.

### 1.9. Elastic Networks in Load-Bearing Connective Tissues

**Figure 7:**
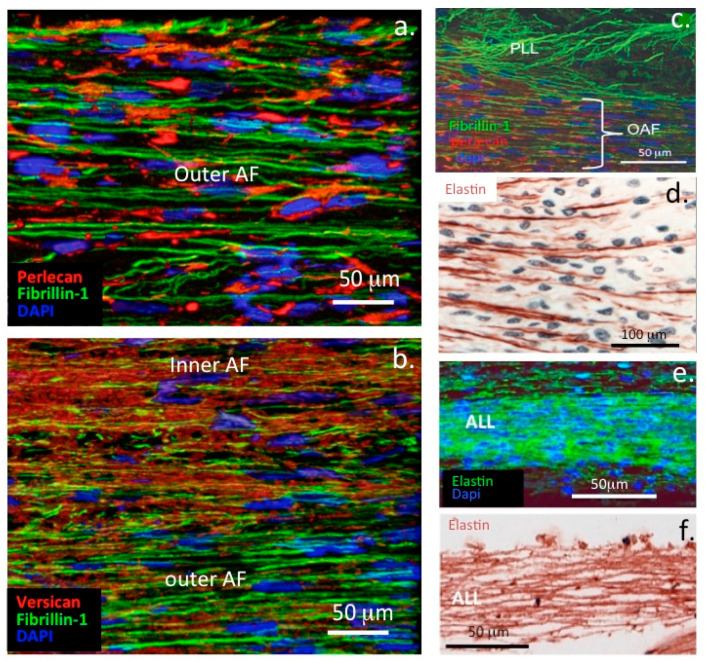
Perlecan interacts with fibrillin-1 and versican in the annulus fibrosus. (**a**–**c**) Confocal immunofluorescence localisations of perlecan (red in **a**,**c**), fibrillin-1 (green) and versican (red in **b**) in the annulus fibrosus of the human foetal intervertebral disc (**a**,**b**) and posterior longitudinal ligament (PLL; cc). (**d**) Immunoperoxidase image shows that elastin fibres act as anchorage points for the attachment of annular lamellae to cartilaginous endplates (**e**,**f**) Elastin immunolocalisation within the anterior longitudinal ligament (ALL) shown by confocal (**e**) and brightfield microscopy (**f**) Cell nuclei depicted in blue. Figures modified from [51,52,53,54] with permission.

As mentioned above, perlecan interacts with a number of ECM components to stabilize tensional and weight bearing tissues such as articular cartilage and IVD. Perlecan interacts with fibrillin-1 and elastin in the AF of the IVD (Figure 7) and in translamellar cross-bridge structures across many lamellar layers [62] (Figure 5). Type VI collagen is also a prominent component of these structures and has been shown to interact with perlecan associated with elastic microfibrils [38,61].

**Figure 8:**
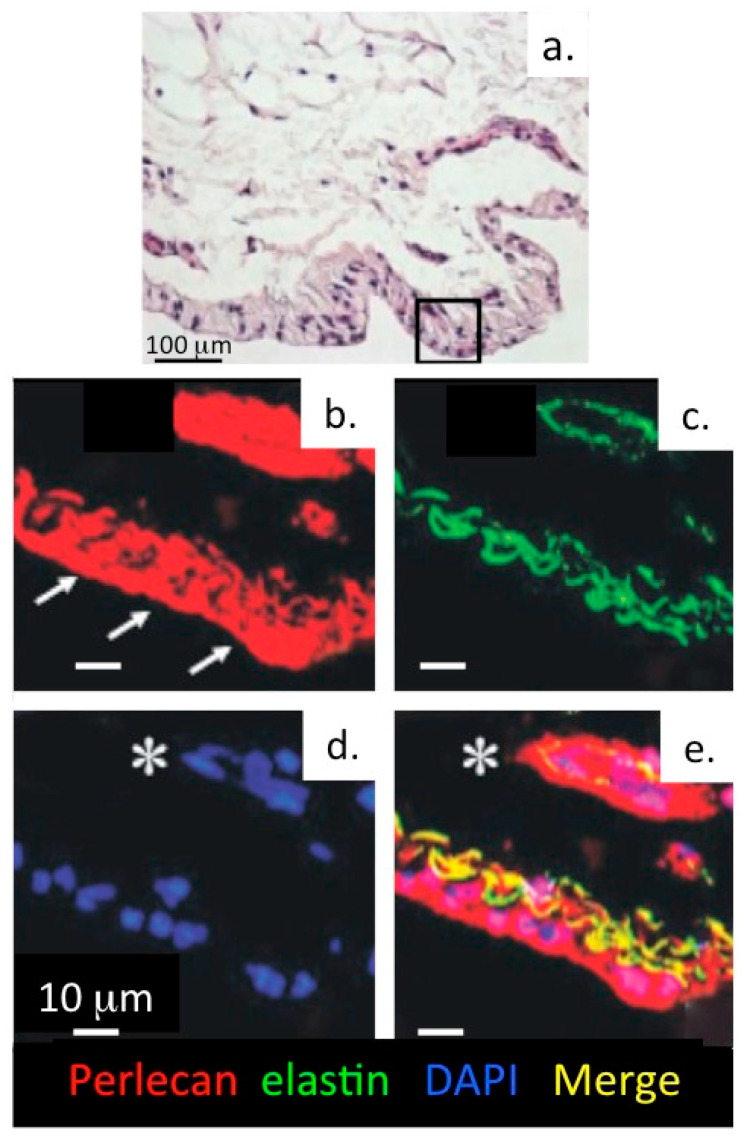
Perlecan and elastin are components of a basal lamina in the knee joint endothelium. (**a**) Haematoxylin & eosin stained tissue section of an 18 month old ovine knee synovium specimen. Confocal localisation of perlecan (**b**, red), elastin (**c**, green), DAPI-stained cell nuclei (**d**) and merged image showing colocalisation of perlecan and elastin (**e**, yellow) within an elastic lamina and surrounding a synovial blood vessel. The arrows indicate the surface of the synovium.The asterisk indicates a blood vessel in the synovium. Figure reproduced from [54] with permission.

While elastin is a well-known component of tissues such as lung and blood vessels, endowing these tissues with the elasticity necessary for their physiological functions, recent research has also revealed the existence of extensive networks of fine elastin fibres in tissues such as articular cartilage and the IVD [115,116]. Microfibrillar elastic networks are evident in bovine and human IVDs. In the outer AF of the IVD, these are organized into parallel microfibril bundles in the interterritorial matrix, co-localized with elastin fibres and aligned parallel to collagen fibres [116]. Fibrillin-1, fibrillin-2 and LTBP-2 are also found associated with these fibrillar networks [117] and contribute to the mechanical properties of these structures (Figure 7). An elastin rich basal lamina is also present in the endothelium of the knee joint (Figure 8).

**Figure 9:**
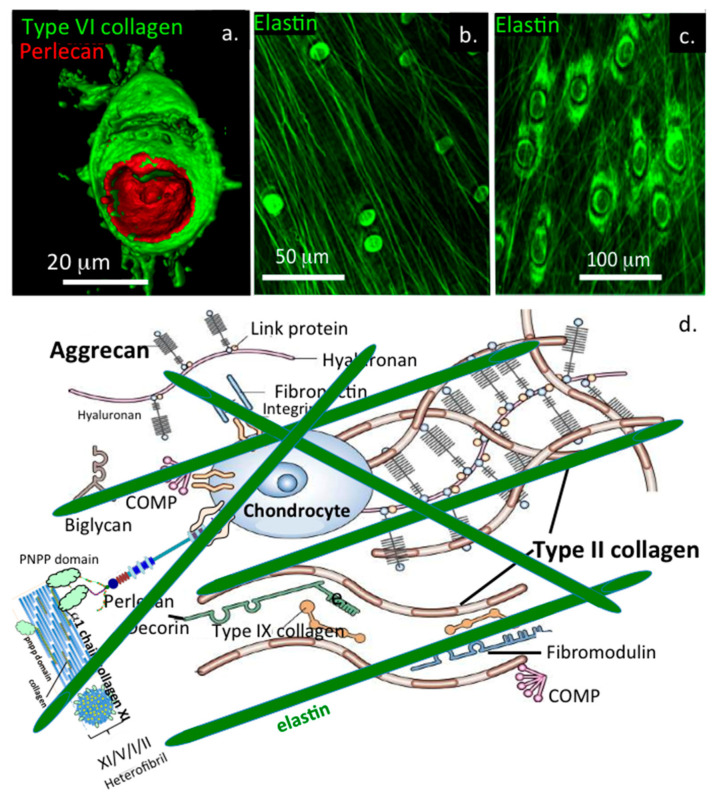
Elastin fibres are components of knee joint articular cartilage. (**a**) Scanning electron micrograph showing the fibrous structure of a chondron surrounding an articular cartilagechondrocyte (**b**) Surface rendered model of a chondron showing the 3D organisation of type VI collagen (green) and perlecan (red) based on confocal data published in [42] (**c**,**d**) Immunofluorescence localisation of elastin (green) within knee joint articular cartilage. Elastin occurs as discrete matrix fibres and as pericellular material focused around chondrocytes. (**e**) Diagrammatic representation of the structural components surrounding articular chondrocytes. Elastin fibres are shown in green. Images a, c, d reproduced from [125] through Open Access. Image e modified [126] under Open Access.

The importance of microfibrillar elastic networks in biomechanical function can be seen across musculoskeletal connective tissues of different organisms and joints. Figure 9 shows elastin fibres distributed in kangaroo knee tibial articular cartilage [118,119]. The kangaroo is a high-performance athletic animal that can maintain a comfortable hopping speed of between 20 to 25 km/h but it can move at speeds of up to 70 km/h over short distances, and can maintain a speed of 40 km/h for nearly 2 km. Thus, the elastic fibres observed in kangaroo knee articular cartilage are expected to make a significant mechanical contribution to the rebound performance of its articular cartilages. Femoro-tibial kangaroo cartilages also have well defined collagen fibre networks which contribute to the high mechanical performance of these tissues [120]. Extensive elastic fibrillary networks are also present in bovine articular cartilage, which is a more sedentary animal [121]. Temporomandibular cartilage also has an extensive arrangement of elastic fibres and these are even found in flexor tendon [122,123,124], a tissue considered to be inextensible and designed for force transmission. Elastic fibres are also major components of the deformable elastic cartilages found in the outer ear (pinna), nose, larynx, trachea, ligaments and vascular endothelium and contribute significantly to their flexibility.

**Figure 10:**
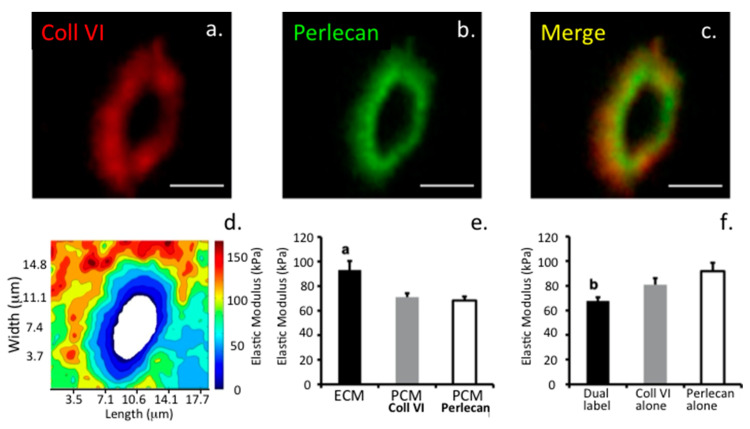
Correlative co-localization of perlecan and type VI collagen with the biomechanical properties of cartilage. (**a**–**c**) Dual immunofluorescence labeling of type VI collagen (**a**, red), and perlecan (**b**, green) within the pericellular matrix compartment of articular cartilage. The adjacent overlay image (**c**) shows their overlapping distributions. Scale bar = 5 μm. (**d**) Contour map of the calculated elastic moduli for the pericellular matrix region shown in (**a**–**c**), as determined by AFM scanning. (**e**) Elastic moduli of cartilage extracellular matrix (ECM) and pericellular matrix (PCM) as defined by the presence of type VI collagen or perlecan. There is no difference between biochemical definitions of the PCM (*p* = 0.70). ECM elastic moduli were significantly greater than PCM moduli (a: *p* < 0.005 as compared to either PCM definition). (**f**) Regions that were dual-labeled for type VI collagen and perlecan exhibited lower elastic moduli than regions positive for type VI collagen or perlecan alone (b: *p* < 0.05 as compared to type VI collagen and perlecan alone regions). Data presented as Mean + SEM. Figure reproduced from [43] with permission.

**Figure 11:**
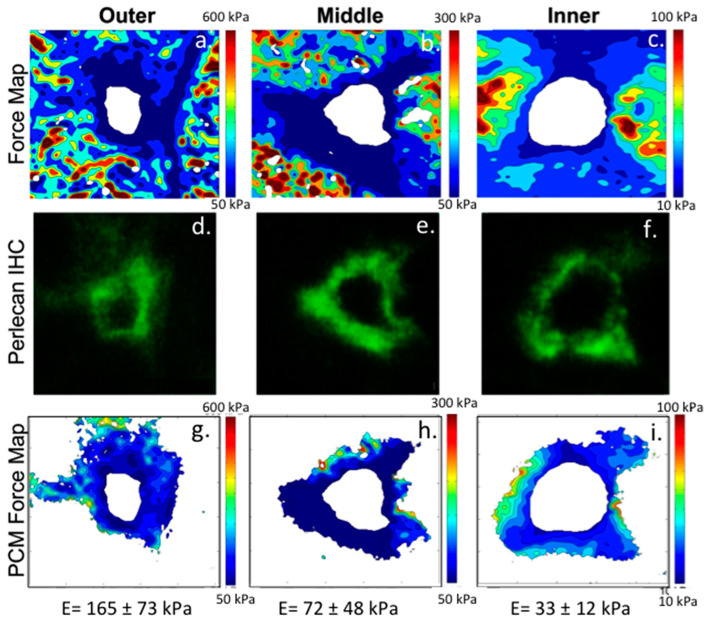
Integration of AFM force maps (**a**–**c**) and fluorescence immunolabeling of perlecan in the meniscus (**d**–**f**). To determine the micromechanical properties of the pericellular matrix (PCM) within the meniscus, meniscal tissue samples were cryosectioned and immunofluorescently labeled for perlecan, which has been shown previously to be localized to this matrix compartment within the meniscus [114]. PCM and ECM properties were evaluated as described previously [43] with PCM sites identified by positive pericellular labeling for perlecan and ECM sites identified by a lack of perlecan staining. Force maps of 20 × 20 µm pericellular regions within each meniscus region (**top** row) were integrated with fluorescent perlecan staining (**middle** row) to define PCM boundaries. The resulting PCM force maps were analysed to yield an average elastic modulus (**g**–**i**). Figure reproduced from [68] with permission.

Correlation of the type VI collagen and perlecan immunolocalization patterns around articular chondrocytes (Figure 10) and meniscal cells (Figure 11) with focal biomechanical data obtained from AFM show that both molecules contribute to the biomechanical functional properties of these tissues (i.e., articular cartilage and meniscus). The elastic fibrils present in these tissues would also be expected to have roles in their resilience and visco-elastic properties, thus complimenting those of perlecan and collagen type VI. This study demonstrates that perlecan and elastin co-localise in many connective tissues and that type VI collagen fibres are frequently deposited parallel to the elastin fibres (Figure 3, Figure 4, Figure 5, Figure 6, Figure 7, Figure 8 and Figure 10). This suggests the likelihood of cooperative interactions between these components in order to facilitate the demanding material properties required to resist tensional and compressive biomechanical loads.

## 2. Conclusions

A vast amount of literature has elucidated perlecan’s roles as a vascular HS-PG. However, with the identification of perlecan in non-vascularised tissues, such as articular cartilage, meniscus, and IVD, its roles in ECM organization and stabilization in tensional and weight-bearing connective tissues has become apparent. ECM networks assembled by cells consisting of perlecan-elastin and perlecan-elastin-collagen type VI assembled by cells are both strong and compliant and contribute to the visco-elastic material properties of tensional and weight-bearing connective tissues. Perlecan is an important component of the PCM and has roles in the assembly and function of structures which connect the cell with its pericellular microenvironment. The PCM is a mechanosensory structure that provides cues to cells which respond appropriately to fluctuations in their microenvironment to maintain tissue homeostasis. Perlecan facilitates the coacervation of tropoelastin and its incorporation with fibrillin-1 in elastic microfibrils at the cell surface. Perlecan also co-localizes with elastin in blood vessels and in the endothelium of synovial joints and has interactive properties with the collagen types VI and XI networks in the PCM of articular cartilage and IVD. Data suggests that perlecan provides a level of compliance to the relatively rigid collagen type VI chondron structure and confers a cytoprotective property. Perlecan is an outstandingly versatile PG in terms of the numbers of physiological processes it regulates and the important functional roles it plays in a variety of different connective tissues. The mechanosensory and neurosensory processes outlined in this review illustrate another chapter in the functional repertoire of this fascinating multifunctional HS-PG.

## Data Availability

All pertinent data are presented within this manuscript.
